# Rare and Common Regulatory Variation in Population-Scale Sequenced Human Genomes

**DOI:** 10.1371/journal.pgen.1002144

**Published:** 2011-07-21

**Authors:** Stephen B. Montgomery, Tuuli Lappalainen, Maria Gutierrez-Arcelus, Emmanouil T. Dermitzakis

**Affiliations:** Department of Genetic Medicine and Development, University of Geneva Medical School, Geneva, Switzerland; Stanford University School of Medicine, United States of America

## Abstract

Population-scale genome sequencing allows the characterization of functional effects of a broad spectrum of genetic variants underlying human phenotypic variation. Here, we investigate the influence of rare and common genetic variants on gene expression patterns, using variants identified from sequencing data from the 1000 genomes project in an African and European population sample and gene expression data from lymphoblastoid cell lines. We detect comparable numbers of expression quantitative trait loci (eQTLs) when compared to genotypes obtained from HapMap 3, but as many as 80% of the top expression quantitative trait variants (eQTVs) discovered from 1000 genomes data are novel. The properties of the newly discovered variants suggest that mapping common causal regulatory variants is challenging even with full resequencing data; however, we observe significant enrichment of regulatory effects in splice-site and nonsense variants. Using RNA sequencing data, we show that 46.2% of nonsynonymous variants are differentially expressed in at least one individual in our sample, creating widespread potential for interactions between functional protein-coding and regulatory variants. We also use allele-specific expression to identify putative rare causal regulatory variants. Furthermore, we demonstrate that outlier expression values can be due to rare variant effects, and we approximate the number of such effects harboured in an individual by effect size. Our results demonstrate that integration of genomic and RNA sequencing analyses allows for the joint assessment of genome sequence and genome function.

## Author Summary

The recent availability of almost fully sequenced human genomes by the 1000 genomes project allows the direct study of genetic variants that influence levels of gene expression in the cell. In this study, we explore the effect of rare and common variants on levels of gene expression. We show that the availability of a more comprehensive list of variants brings us closer to the likely causal variants, and we discuss their genomic and evolutionary properties. We also demonstrate the effects of variants that change splicing patterns or length of the protein product, the putative joint impacts of variants that affect gene expression, and those that affect protein structure. Finally, we show the impact of rare regulatory variants that cannot be detected by the conventional methodologies of association and require the interrogation of full genome sequencing and full transcriptome sequencing. These approaches bring us closer to the implementation of these data and methodologies to a direct clinical application.

## Introduction

Deeper characterization of genetic variation is becoming increasingly available with advances in DNA sequencing technology [1]–[5]. This improves our ability to pinpoint protein-coding variants which disrupt protein structure, and has already begun to provide insight into the genetic basis of disease with unknown etiology [6], [7]. In addition to protein-coding variation, access to a more complete spectrum of genetic data facilitates the discovery of regulatory variants. However, relative to protein coding variation, the information about the structure of gene regulatory architecture is incomplete and the existence of a regulatory variant is largely inferred through its association with gene expression. Such associations have previously been identified as exhibiting widespread and tissue-specific patterns [8]–[11]. They are also increasingly linked to the basis of human phenotypic diversity [11]. For instance, recent studies have implicated the role of regulatory variants in the etiology of diseases such as obesity [12], celiac disease [13] and migraine [14]. Now, the compendium of variants acquired from genome sequencing of population samples provides increased potential for uncovering new associations, many of which, given this enhanced resolution, are presumed to be causal. Furthermore, we are able to begin to analyse genome-wide signals of interactions between disruptive protein-coding variation and regulatory variation. We investigate the landscape of regulatory variation as surveyed by population-scale sequencing by using data acquired from the 1000 genomes project, together with gene expression data in 60 CEU individuals (CEU: Utah residents with ancestry from northern and western Europe) acquired using RNA sequencing (RNA-Seq) and 57 CEU and 56 YRI individuals (YRI: Yoruba in Ibadan, Nigeria) acquired using gene expression arrays [15], [16]. In this study, we demonstrate the value of almost complete information from the 1000 genomes project to reveal the fine structure of rare and common regulatory variation.

## Results

### eQTL discovery

**Figure 1:**
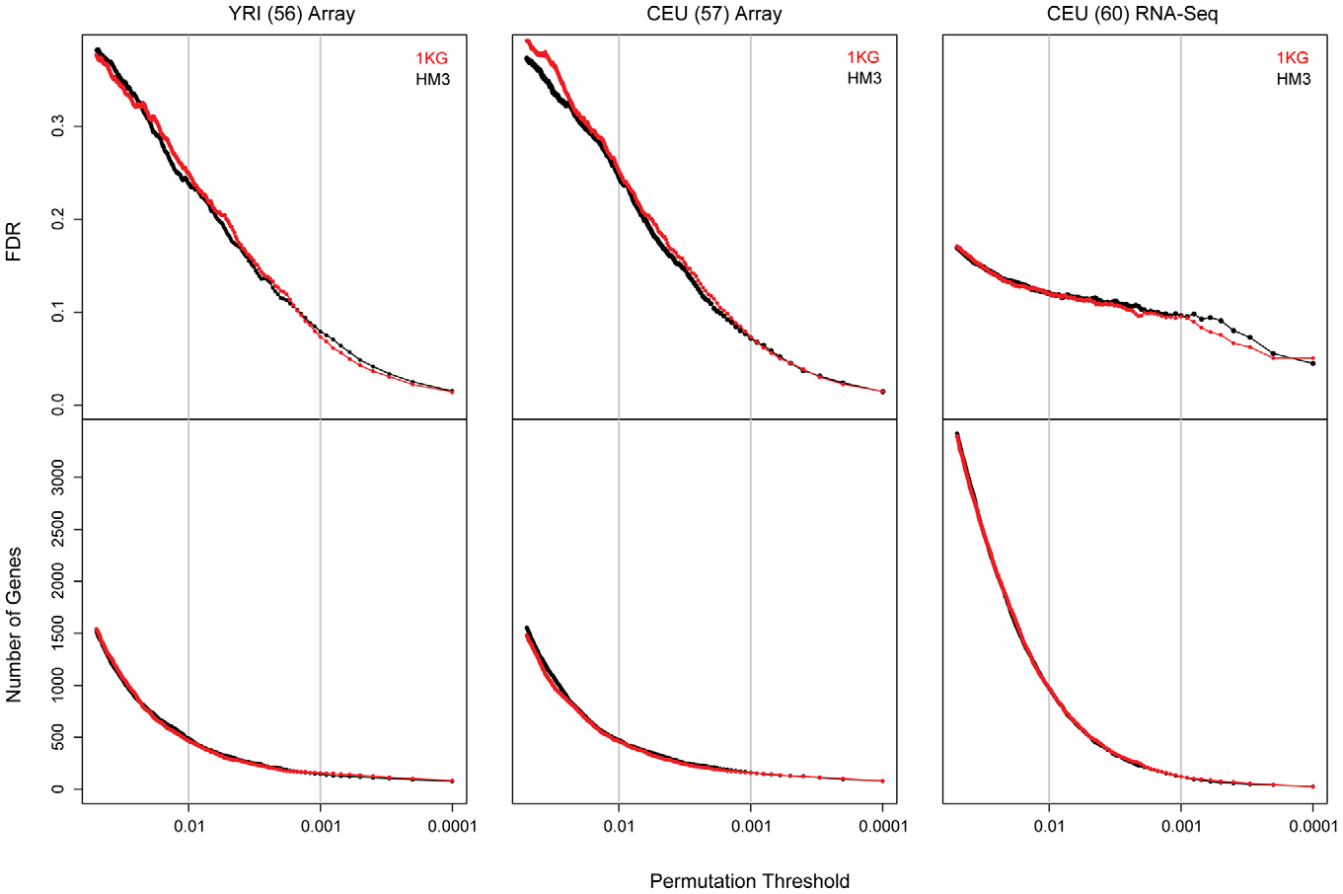
Comparison of eQTL discovery in HapMap 3 and 1000 genomes project data. We compared the discovery of eQTLs from HapMap 3 (black) and 1000 genomes (red) project variants by expression platform (LCL expression interrogated on arrays from 56 Africans and 57 Europeans, and by RNA sequencing of 60 Europeans) across log-mean permutation threshold. At all levels of FDR across the permutation threshold range, we observe similar levels of detection of eQTL genes between HapMap 3 SNPs and 1000 genomes project SNPs. This indicates that given our sample sizes, similar levels of regulatory haplotypes are recovered despite the 5–7× increase in the number of common variants from DNA-sequencing. Comparison relative to observed p-value instead of FDR (Figure S1) accentuates the effect of increased number of tests in the 1000 genomes project data. Furthermore, the comparison between array and RNA sequencing data shows a reduction in the FDR relative to the total number of genes for relaxed permutation thresholds, indicating improved performance of the platform to uncover eQTLs in this FDR range.

We assessed the number of expression quantitative trait loci within 1 Mb of annotated genes (*cis*-eQTLs), and compared the power of HapMap3 (HM3) against the much higher SNP density of the 1000 genomes project (1KG) genetic variants, using gene expression data from lymphoblastoid cells for matching individuals (see Materials and Methods). For both CEU and YRI, similar numbers of eQTLs were found between the two projects independent of FDR (estimated by permutations; Figure 1 and Figure S1). This suggests that, with given power, the majority of the common regulatory effects can be captured by genome-wide SNP arrays. Using RNA sequencing data, we were also able to survey the difference between 1KG and HM3 for regulatory variation detected through allelic imbalance of heterozygous coding sites. In 1KG, twice as many heterozygous sites (36015 versus 14281) had significant allele specific expression (ASE; reviewed [17]) effect (p≤0.05), corresponding to 4971 genes versus 3175 genes. The median log effect size for this imbalance was 1.39 (Figure S2). This increase provides more power to explore within individual regulatory variation.

**Figure 2:**
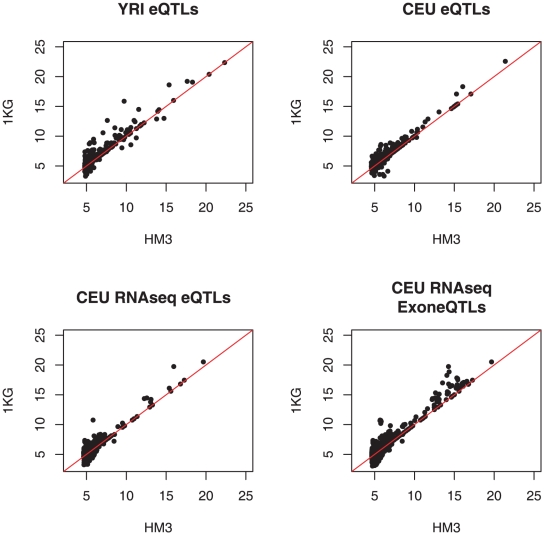
Fine mapping of HapMap 3 eQTLs into 1000 genomes variants. For eQTLs discovered with HapMap 3 (HM3) variants we assessed the best p-value of a variant in linkage disequilibrium (D′≥0.8) with the HapMap 3 variant in the 1000 genomes (1KG). This discovery was compared for all populations and expression platforms and in between exon and gene eQTLs for the RNA-Seq data. We found that usually a better association was uncovered in 1KG, suggesting that we are more likely to be observing the causal variant. For CEU-eQTLs (top right panel) discovered using arrays, 189 of 398 associations were better in the 1000 genomes (only 34 worse). For YRI-eQTLs (top left panel) discovered using arrays, 187 of 427 associations were better in the 1000 genomes (28 worse). For CEU gene and exon-eQTLs discovered with RNA-seq, 362 of 821 were better in the 1000 genomes (129 worse) and 1130 of 2598 were better (371 worse), respectively.

Given that the 1KG data provides an almost complete ascertainment of common SNPs, we sought to assess whether we are more likely to detect potentially causal regulatory variants. We observed that nearly 80% of all eQTVs discovered with the 1KG were not discovered with the HM3 (Table S1). This indicates that if these new variants are bonafide causal variants, whole genome sequencing is uncovering a large number of previously unidentified variants. Conversely, however, for the eQTVs discovered with the HM3, up 65% would not have passed the discovery threshold in the 1KG due to the extra multiple testing correction implicit through having 5–7 times as many variants (Table S2). In order to investigate if the 1KG eQTLs exhibited characteristics indicative of being a functional variant, we fine mapped HM3-discovered eQTLs into the 1KG (see Materials and Methods). We observed that for both populations, independent of the gene expression platform, the majority of these HM3 eQTLs were found in the 1KG with the same or a different variant of higher significance, and infrequently (<16%) would the association be worse, likely due to genotyping errors in the 1KG (Figure 2). Next, we compared the properties of these eQTLs in HM3 and 1KG. Since previous analyses have identified a strong enrichment of eQTLs around the transcription start site [18]–[20], we investigated if the 1KG associated SNPs were more proximal to the transcription start site of their associated gene than the HM3 associated SNPs, but no significant trend was observed (Figures S3, S4 and S5). This supports recent observations that the strongest effects on gene expression are not exclusively defined through promoter variation [15], [21]. We next asked if the newly discovered variants were on more evolutionarily conserved sites, which would suggest that they are more likely to be causal variants [22]. In this analysis we had to account for the fact that the HM3 SNPs are more conserved overall (Student's T-test p<2e-16; MW p<2e-16). Thus, we compared the within platform difference between the best association and the second best-linked association, expecting that the increase in conservation between the two could be higher within 1KG due to the best association being more often the causal variant than in HM3. However, no significant difference was observed (Figure S6), which indicates, consistent with ENCODE findings, that many regulatory elements and thus also genetic variants in these elements are unconstrained [23], [24].

### eQTLs for alternative splicing

We investigated the allelic expression properties of transcript variants that have a putative impact on transcript structure. For splicing variants (MAF≥5%), we saw an enrichment in associations in RNA-Seq data for the respective donor and acceptor exon read count levels (the number of RNA sequencing reads which overlap the exon for an individual, see Materials and Methods) compared to a background derived from synonymous variants (Figure S7). This enrichment was observed independent of mapping quality filter confirming that it is not due to mapping biases (Figure S8). We also investigated gained-stop codon variants for signals of nonsense mediated decay, finding greater than 4-fold enrichment in exon read count level associations for overlapping exons when compared to synonymous and nonsynonymous variants (Figure S9). When assessing this enrichment separately through ASE signals in the RNA-Seq data, we found that 44% (66 of 150 testable heterozygotes at 32 sites) of stop gained variants where ASE can be detected are significant compared to only 18.8% of synonymous variants and 20.9% of nonsynonymous variants.

### Cis-regulatory modifiers of protein-coding variants

Regulatory variation can also modify the functional impact of protein-coding variation. We had previously reported that 18.2% of nonsynonymous variants were modulated by regulatory variation [25]. We now discovered that at least 20.9% of testable heterozygotes for nonsynonymous SNPs (n = 32859) had significantly different expression levels of the two nsSNP alleles (p≤0.05; this is 23.3% for n = 38645 when both known alleles are not required to be observed). This corresponds to 46.2% of nonsynonymous variants having an ASE effect in at least one individual (n = 5686). These results suggest that regulatory variation may have a fundamental role in explaining individual differences in penetrance of disease predisposing variation. Thus, surveys of coding variation through large-scale exome resequencing studies [7], [26]–[28] would benefit from complimentary information of regulatory variation e.g. from RNA sequencing of the same samples.

Given that regulatory modifiers of protein-coding variation were prevalent, we looked for population genetic signals of interaction between protein-coding and *cis* regulatory variation. Such co-evolution would imply a selective advantage of some regulatory and coding variant combinations over other haplotypes in that locus potentially increasing linkage disequilibrium (LD). In order to seek for such patterns from the ASE data, we calculated the proportion of heterozygous individuals that have significant ASE as a proxy for linkage disequilibrium between the coding ASE variant and the unknown regulatory variants. Furthermore, we used both HM3 and 1KG datasets to control for putative effects of genotyping error (Figure S10). We observed an increase signal for nonsynonymous compared to synonymous variants (p = 3.5e-4; Figure S11), which is suggestive coevolution of functional nonsynonymous and regulatory variants. The result is unlikely to be caused by nonsynonymous SNPs being causal regulatory variants more often than synonymous SNPs: the two types of variants have a nearly equal enrichment of exon associations (Figure S9), and while a change in protein structure might change the overall expression level of the gene itself through an autoregulatory mechanism, this is not expected to lead to allelic imbalance. When stratifying by the derived allele's expression, in the 1KG data we observed a large and statistically significant enrichment of the ASE proportion for rare nonsynonymous variants where the derived allele has lower expression than the ancestral (Figure S12). This suggests that some low-frequency deleterious coding variants may be tolerated in the population only because they lie on a lower expressed haplotypes and thus have reduced penetrance. This may be particularly important for understanding the phenotypic effects of loss-of-function variants – it has been estimated that each person carries 250 to 300 loss-of-function variants (50 to 100 of which are previously implicated in inherited disorders) [29], but sometimes their functional impact may be diminished or strengthened by their regulatory background.

### Detection of rare regulatory variants

**Figure 3:**
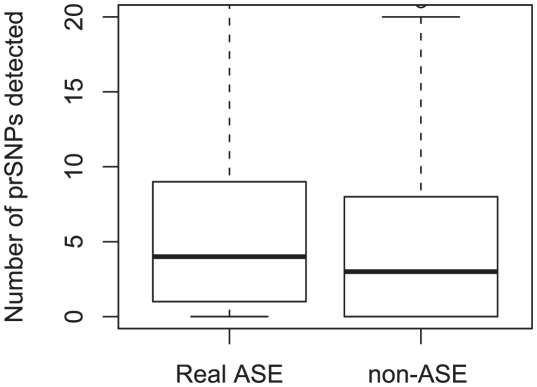
prSNPs detected for rare ASE effects (real and non-ASE). For each ASE effect, we observe more prSNPs for the real ASE versus the non-ASE null data. On average, we find 1 more prSNP in the real ASE data, which is expected given that the real data should contain at least 1 causal variant more than the null.

Genome sequencing offers the ability to interrogate the functional impact of recent and rare regulatory variants in individuals [30]. We calculated whether individuals sharing an rare ASE effect are more likely to show increased haplotype sharing, measured as haplotype homozygosity, which would be a signal of the ASE effect being driven by a shared rare regulatory variant (as described in [15]). Concordant with previous results, we found an excess of haplotype sharing for rare ASE haplotypes (Figure S13). Next, we sought to identify the putative causal variants by investigating genetic variants which were perfectly concordant with this rare ASE effect (see Materials and Methods). For each such effect, we identified a median of 4 and a mean of 8.83 putative regulatory SNPs (prSNPs) within 100 kb of the TSS (compared to a median of 3 and mean of 7.64 putative regulatory SNPs under the null; Figure 3 and Figure S14). Altogether, we identified at least one putative regulatory SNP for 1711 of 2693 genes demonstrating a rare ASE effect (compared to 1517 under the null), totalling 23234 prSNPs for rare ASE effects (compared to 20393 under the null). Additionally, the prSNPs showed signs of increased functional potential compared to the null group: they were more likely to be distributed around the transcription start site and within the gene relative to control SNPs (*χ* ^2^ p-value<2e-16; Figure S15). Furthermore, the prSNPs were more likely to have a lower derived allele frequency (p = 3.437e-12) and also trended to have higher evolutionary conservation, indicating that they are more likely to be functional and putatively slightly deleterious (p = 0.07 with PhyloP vertebrate conservation scores; Figure S16).

**Figure 4:**
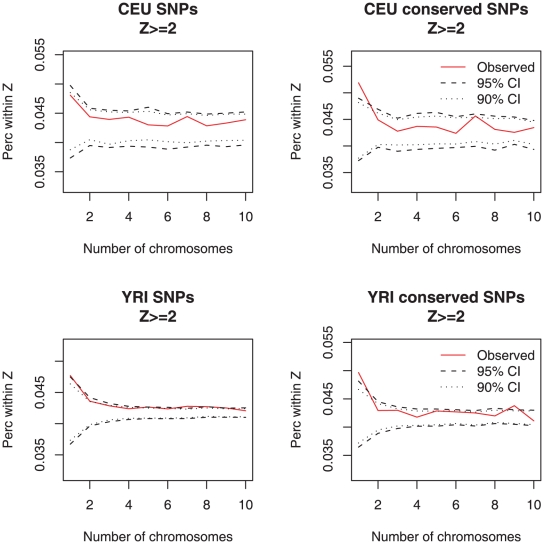
Excess of rare regulatory variants coincident with expression outliers. We calculated the excess of expression outliers as a function of frequency for all SNPs within 100 kb of the transcription start site of array-quantified genes for both Europeans and Africans. We further sub-selected to only include SNPs in 17-way most conserved elements from UCSC. We observed an enrichment of conserved singleton SNPs coincident with expression outliers (Z>2; p<0.05). The confidence intervals were estimated by randomizing expression labels 200 times.

We then sought for a signal of rare regulatory variants underlying large changes in gene expression by calculating whether individuals with outlier array expression values are enriched for rare genetic variants. We found that individuals with gene expression Z-score ≥2 (a measurement of how far the observed value is from the mean of the sample – see Materials and Methods) have an excess of rare variants within 100 kb of the transcription start site, a signal that was statistically significant (outside the 95% CI) for rare variants landing in highly conserved sites derived from 17-way vertebrate alignments (Figure 4). The average log-2 difference in expression from the mean for these variants was 0.74±0.52 in CEU and 0.66±0.49 in YRI (Figure S17). Overall, there was an excess of 162 coincident singleton, conserved SNPs with expression outliers (Z≥2) in the CEU sample (one-sided p-value<0.05) and the same number, 162, in YRI (one-sided p-value<0.05). Divided by the number of studied individuals, this indicates that there are approximately 3 such effects per individual for this cell type. For other Z-score thresholds and for RNA-Seq data, we observed the same type of enrichment (Figures S18 and S19).

## Discussion

In this study, we have analyzed common and rare regulatory variation in the human genome using resequencing data, highlighting the many advantages of population-scale sequencing in understanding the spectrum of functional variation in the genome. The comparison of eQTL discoveries using 1000 genomes and HapMap 3 data indicated that while many novel associations are discovered with resequencing data, most of common effects are already captured with genotyping arrays. Even though mapping common causal regulatory variants remains a challenge, we observed a clear enrichment of regulatory effects in splice-site and nonsense SNPs. Furthermore, we showed that regulatory variation can putatively modify the effects of a large proportion of nonsynonymous coding variants, and present population genetic evidence suggestive of such interactions. The possibility to study rare variants has been one of the main motivations for large-scale resequencing experiments, and we presented several novel approaches to analyse rare regulatory variation from genomic as well as RNA sequencing data. For rare regulatory effects identified from RNA sequencing data, we were able to pinpoint a median of four putative regulatory variants per rare effect, one of which is expected to be causal – a number low enough for feasible experimental validation. Additionally, individuals with outlying expression values were shown to have an enrichment of rare conserved regulatory SNPs, with each individual carrying an estimation of approximately 3 rare regulatory variants that have a large effect (Z> = 2) on gene expression in the studied cell type. Across all the tissues and developmental stages, each individual is expected to have even hundreds of such rare, large effect regulatory variants. We have also demonstrated how studies integrating genomic or exome sequencing with RNA sequencing data from different tissues will also provide information of how the functional effects of protein-coding variation are modified by regulatory variation. Altogether, these approaches will bring us closer to a joint assessment of how genome sequence affects genome function, and how this relates to phenotypic diversity.

## Materials and Methods

### SNP and indel genotypes

We used 1000 genomes polymorphisms from the March 2010 pilot 1 and 2 release (www.1000genomes.org; REL-1003) and HapMap 3 release 3 genotypes (www.hapmap.org). For association analysis, we used 5 404 174 common (MAF≥0.05) SNPs for 60 RNA-sequenced CEU individuals (Utah residents with ancestry from northern and western Europe) and 5 329 982 and 6 976 232 SNPs for 57 and 56 expression-arrayed CEU and Yoruban individuals (Yoruba in Ibadan, Nigeria), respectively. For two individuals which were parents in a CEU trio which had variants independently called in the 1000 genomes (pilot 2), we intersected their genotype calls with pilot 1 calls; in cases where no genotypes were reported in the trio individuals, we added the reference homozygote state. Between pilot 1 and pilot 2, 3 398 517 sites were concordant and had genotypes reported (for 950 sites the reference and alternative allele were different between the trio individuals and the 58 pilot 1 individuals and these sites were excluded from further processing). For indels, we used calls from the same release. In total, 592 081, 586 604 and 710 931 common indels were used in each population sample (60 CEU with RNA-Seq, 57 CEU with arrays and 56 YRI with arrays). For rare variant and ASE-based analyses we used only the pilot 1 1000 genomes genotype calls; this was to prevent biases due to the improved rare variant calling on the pilot 2 trio.

### Gene annotation and gene expression data

RNA-sequencing and expression arrays experiments were performed and quantified on RNA extracted from lymphoblastoid cell lines as previously reported [15]. We updated our annotation for RNA-Seq quantification to use the Gencode v3b annotation [31].

### Exon read level associations

For RNA-Seq data, we calculated associations per exon by quantifying the number of reads overlapping known exon annotation for each individual and then performing Spearman rank correlation with respect to corresponding genotypes as previously reported [15].

### Allele-specific expression analysis

Allele-specific expression was assessed by calculating the allelic imbalance of variants over heterozygote positions. Significance is assessed using the binomial probability distribution where the probability of success is weighted by that individual's/lane's reference allele to non-reference allele mapping bias. ASE variants used in this study were not monoallelic as we required both reported alleles to be observed at least once. We also conditioned on the ASE effect being present for reads quantified above MAQ10 mapping quality and Phred score of 10 but subsequently reinforced there was no threshold effect by requiring significance when there was no mapping or base quality filter.

### Fine mapping HapMap3 eQTLs into the 1000 genomes

The best association per gene (or in the case of RNA-Seq data per exon) at or below the 0.01 permutation threshold was fine-mapped from the HM3 into the 1KG data. Each of these eQTL variants from the HM3 was compared to D′ calculated by Haploview for all the 1KG variants with a Spearman association of p≤10e-3 with the same gene. The 1KG variants which were in LD (D′≥0.8) with the original HM3 variant were deemed to be underlying the same effect originally discovered in the HM3; the best association for that gene in the 1KG meeting this LD criterion was selected for comparison to the original HM3 variant. This methodology allowed us to survey new discoveries irrespective of whether they were the same variant, different variants at different frequencies or divergently-located with respect to the transcription start site.

### Functional variation determination

Functional variation was determined using the EnsEMBL 54 pipeline [32]. Splicing variants were compared to the Gencode annotation and were deemed accurate for essential splicing variants if they were within 5 bp of an exon boundary and accurate for a general splicing variant if they were within 100 bp of an exon boundary. For testing exon association enrichment, we took the splicing variant associations for their respective donor (5′) and acceptor (3′) exons. To find a matching set to test for enrichment of association, we considered synonymous variants which were greater than 15 bp away from an exon boundary. We calculated enrichment by calculating the qvalue statistic1-π0 for acceptor and donor associations only when there were more than 30 associations; the log-ratio of this enrichment was reported [33]. This calculation was made across the range of mean read depths for exons from 1–1000 reads. Stop gain variants were tested against the exon they overlapped and were also compared in a similar way to synonymous and nonsynonymous mutations.

### Conservation and allele-frequency analysis

The PhyloP base-wise conservation scores were based on alignments of 46 vertebrate genomes, 33 mammalian genomes, and 10 primate genomes, and were downloaded from UCSC [34]. Ancestral alleles were obtained from the 1000 genomes pilot release.

### Haplotype homozygosity from RNA-Seq data

Haplotype homozygosity indicates the relative age of a haplotype by assessing the incidence (or lack thereof) of recombination or other mutation. This is achieved by comparing the extent of homozygosity between haplotypes by calculating the length of sequence from a target marker until a mismatch occurs. Phased data is required in non-haploid species to assess and compare individual haplotypes from a target marker position. Furthermore, since haplotype homozygosity is being assessed from a heterozygous target marker (required for assessing ASE), a decision needs to be made about what allele should be taken to represent the reference haplotype and in what direction haplotype homozygosity should be assessed (5′ or 3′). Here, we used phasing data as provided by the 1000 genomes project pilot release and compared all haplotypes carrying each allele for the heterozygous marker and in both directions to select the allele and direction with the average longest tract of haplotype homozygosity. Then, given this direction and reference haplotype, we take as a criterion for comparison that there must be at least 6 individuals where between 2 and 4 have ASE significant haplotypes in the same direction and at least 2 are non-significant for ASE. To compare the extent of haplotype homozygosity given the reference haplotype and the ASE status of each haplotype we compare significant ASE to significant ASE haplotypes and significant to non-significant ASE haplotypes and compute the average length of haplotype homozygosity for all pairwise individual comparisons within these categories. We then stratify the results for each ASE marker based on the number of significant ASE haplotypes were original discovered.

### Causal regulatory variant detection for rare regulatory haplotypes

To identify putative regulatory SNPs on rare regulatory haplotypes using ASE calculations we looked for all variants within 100 kb of the transcription start site which satisfied ASE sharing in 1, 2 or 3 individuals when at least 6 heterozygotes individuals could be tested for ASE. To satisfy sharing, the variant must be heterozygous with the same direction of effect (assessed through phasing) when an ASE effect is present in an individual and homozygous when the ASE effect is not present. To assess how well our putative causal regulatory variants discovery was performing we assessed the distribution of discoveries around the transcription start site by comparing counts of real predictions versus control predictions in 5 kb windows using the Fisher's exact test (Bonferroni-corrected for multiple testing). Control (null) predictions were obtained by matching each ASE test by reassigning significance in the opposite direction. For instance, if there were 6 heterozygotes, 2 of which show ASE, the control reassignment would assign ASE to the two least significant heterozygote individuals. To assign direction of effect, we matched the distribution of real directions determined with the phasing data to the control set.

### Z-score analysis

We compared the co-occurrence of expression outliers with rare variants by recomputing our expression files as Z-scores and binning at each allele frequency the distribution of expression measurements that were incident with the non-reference variant. To assess significance of divergence in this distribution, individual labels were permuted 200 times and 90 and 95% CI were obtained.

## Supporting Information

Figure S1 Comparison of HapMap 3 and 1000 genomes project eQTL discovery (p-value thresholds). The discovery of eQTLs from HapMap 3 (black) and 1000 genomes (red) project variants by expression platform (LCL expression interrogated on arrays from 56 Africans and 57 Europeans and LCL expression from RNA sequencing of 60 Europeans) was compared across p-value thresholds. Here, the equivalent permutation threshold for the 0.01 and 0.001 levels are highlighted. This highlights that the addition of 5–7× as many markers through whole genome sequencing is requiring a higher threshold for association significance providing the potential to remove many weaker but previously significant regulatory haplotypes.
(EPS)

Figure S2 Fold changes for significant allele specific expression variants. We plotted the fold change distribution for significant ASE variants (p≤0.05). The median fold was 1.39. The mean inter-individual fold change was 1.41 with a standard deviation of 0.16.
(EPS)

Figure S3 Comparison of distance to transcription start site of fine mapped variants from HapMap3 (HM3) into 1000 genomes (1KG). An estimate of whether newly discovered associations in the 1KG are more likely to be causal mutations is to assess their position relative to the transcription start site (TSS) with the expectation that causal mutations may be more proximal to the TSS. We assessed this for array-based and RNA-seq eQTLs and for each platform we observed that there was no significant trend towards to the TSS for improved associations. However, we observed that many of the newly discovered variants are proximal to the previous reported association from HM3. This supports that measured eQTLs are not strictly limited to a class that is influencing the proximal promoter machinery.
(EPS)

Figure S4 Comparison of distance to exon midpoint of fine mapped variants from HapMap3 (HM3) into 1000 genomes (1KG). Considering that many newly discovered associations for the 1KG may be marking genetic differences in splicing or transcript termination, we interrogated if these associations were more likely to be discovered proximal to the mid-point of the exon compared to the original associated variant discovered in HM3. We assessed the distance to the exon midpoint for exon-eQTLs discovered in HM3 that were also discovered and in the 1000 genomes. We did not observe a pattern which preferentially indicated that the new associations were closer to the exon midpoint. This indicates that in general the improvement in association is not a function of a particular component of gene regulatory architecture but is likely a heterogeneous mix of different genetic effects which influence the transcriptome.
(EPS)

Figure S5 Comparison of distance to transcription start site of fine mapped variants from HapMap3 (HM3) into 1000 genomes (1KG) for CpG versus non-CpG promoters. We hypothesized that the genetic effects on gene expression would be manifested differently depending on whether a gene's proximal promoter contained a CpG island. Our rationale was that CpG promoters might have broader effect locations while non-CpG and likely more binding-sequence dependent promoters might be constrained closer to the TSS. We did not observe that either had preferential discovery closer to the transcription start site. A prevailing observation was that discovered effects were close to the original tagging variant in HM3.
(EPS)

Figure S6 Comparison of conservation difference between best association and second best association within platforms. Given that the average conservation for HM3 variants was higher than in 1KG (Student's T-test p<2e-16; MW p<2e-16), we tested if we saw a larger improvement in conservation score between the best association and the second best association (conditioned on them being in D′≥0.8). This was matched for the same eQTL as the HM3 and 1KG best associations were also in D′≥0.8. In general, this was not observed except for YRI (and there only for primate and not mammalian or vertebrate conservation differences). This suggests that many of these newly discovered associations in 1KG are not more highly constrained at the base level then the HM3 associated-variants.
(EPS)

Figure S7 Enrichment of 1000 genomes splicing associations. To assess the impact of splicing variation identified through annotation, we assessed the enrichment of associations that we observed for acceptor versus donor exons stratified by acceptor (left-panel) and donor snps (right-panel). We plotted this relative enrichment as a function of mean number of reads per individual to assess if difference in gene quantities were responsible for differential discovery of splicing effects.We further also plotted as the background the same relative enrichment for synonymous variants which were greater than 15 bp away from the splice junction and had not be identified as splicing SNPs. We observed that we could detect enrichment of splicing variation in the RNA-seq data when sufficient reads were present. This was consistent specifically for better quantified exons (with sufficient read depth). This pattern of enrichment for splicing variants is reinforced by both tests having consistent deviation from the background despite some observable stochasticity. In this plot, for each binned measure of enrichment at least 30 variants were available. Enrichment was measured as the log ratio of the 1-pi0 statistic for acceptor versus donor exon associations (using q-value statistics).
(EPS)

Figure S8 Enrichment of 1000 genomes splicing associations without mapping filter. To address if the enrichment of splicing variants we observed was due to threshold effects due to mapping we removed mapping quality filters and repeated the same analysis. For both acceptor and donor variants we observed the same enrichment relative to synonymous variants.
(EPS)

Figure S9 Enrichment of exon-eQTLs for overlapping exons of synonymous, nonsynonymous and gained stop variants. We observed significant enrichment for exon-eQTLs for gained stop variants when compared to enrichment we observed for synonymous and nonsynonymous variants. This enrichment suggests that nonsense mediated decay is detectable in RNA-Seq data. The level of enrichment was estimated using the 1-pi0 statistic.
(EPS)

Figure S10 Proportion of ASE of all heterozygotes per variant with respect to derived allele frequency. To investigate the relationship between regulatory and coding variation we looked at the proportion of heterozygotes which had allele specific expression (ASE). This proportion is expected to be high when the coding variant is in strong linkage with a cis-regulatory variant causing the allelic imbalance. We calculated the proportion of ASE with respect to DAF for cSNPs with ASE and at least 3 heterozygotes in the full 1KG data (a), in 1KG SNPs with both allele observed at least once (b), and in SNPs with genotypes concordant with HM3 data (c). The dark blue lines are means in sliding windows of 600, 400 and 150 SNPs respectively, proportionally to the number of SNPs, and the cyan lines denote the 10% upper and lower quantiles. The analysis was performed for these three datasets to control for possible effects of false heterozygote genotype calls in the 1KG data. However, it is frequent that only one allele is observed in the RNA-Seq data even when analyzing the high-quality heterozygotes from the HM3 dataset (2042 of 6859 (30%) of ASE heterozygotes). This proportion was higher in the full 1KG data (41%, 21122/51585), which could be caused by genotyping error. However, false ASE calls are likely to create single random ASE observations per SNP and decrease the mean ASE proportion; thus, our analysis looking of an increase is likely to be conservative in this respect. Altogether, the results show increased ASE proportion in the end of the DAF distribution. This can arise by chance through new regulatory mutations landing on haplotypes carrying a rare coding allele, or a new coding mutations landing on haplotypes carrying a rare regulatory allele. Additionally, even co-evolutionary pressure on a haplotypes containing both variants can increase the proportion of ASE.
(EPS)

Figure S11 Proportion of ASE of all heterozygotes per variant for synonymous and nonsynonymous variants. For sSNPs and nsSNPs with ASE and at least 3 heterozygotes in the full 1KG data (a), in 1KG SNPs with both allele observed at least once (b), and in SNPs with genotypes concordant with HM3 (c), we observed that that the proportion of ASE is higher for nonsynonymous than synonymous variants. This may suggest increased LD between regulatory variants and nsSNPs through favoring of functional coding variants on a particular regulatory haplotype over other haplotype combinations. Significance was assessed using Mann-Whitney.
(EPS)

Figure S12 Mean proportion of ASE of all heterozygotes with respect to derived allele frequency for synonymous and nonsynonymous variants where the derived allele is higher or lower expressed. The proportion of heterozygote individuals with ASE was separated by derived allele's expression (either “loss” or “gain”) for sSNPs and nsSNPs with ASE and at least 3 heterozygotes in the full 1KG data (a), in 1KG SNPs with both allele observed at least once (b), and in SNPs with genotypes concordant with HM3 (c). The lines are means in sliding windows of 300, 200 and 75 SNPs respectively, proportionally to the number of SNPs, and the Mann-Whitney p-values for sSNPs versus nsSNPs have been calculated in DAF bins of 0.05 with an overlap of 0.025. The proportion of ASE is high especially in rare nonsynonymous variants where the derived allele is lower expressed, potentially suggesting evolutionary preference of rare derived nsSNP alleles to be on the lower expressed haplotype. This could be a signal of rare deleterious coding variants that are tolerated in the population due to being on a lower expressed haplotype, which lowers their penetrance. The trend can be seen also in the 1KG data with both alleles observed but is expectedly weaker, since this phenomenon would be strongest when the rare derived allele is hardly expressed at all. The lack of signal in HM3 may be explained by these variants being underrepresented in the SNP genotyping arrays.
(EPS)

Figure S13 Extent of haplotype homozygosity for shared ASE effects. To identify signals of recent and rare eQTLs in the 1000 genomes data, we assessed the extent of haplotype homozygosity for shared ASE individuals versus ASE and non-ASE individuals (when there was greater than 6 heterozygotes individuals which were quantifiable for ASE and a significant ASE effect was observed in 2,3 or 4). We observed that when ASE was shared among 2 or 3 individuals, there were longer tracks of haplotype homozygosity indicating shared genealogy for an underlying causal mutation. This result supports previous findings we observed using HapMap3 phasing data (Montgomery et al, 2010).
(EPS)

Figure S14 Number of prSNPs detected for real ASE and non-ASE effects. We plotted the distribution of the number of real prSNPs from control prSNPs detected for each ASE effect. Here, at each testable marker, the number of real prSNPs is subtracted from the number of control prSNPs identified and plotted as a histogram. The shift to the left shows that many more ASE effects have more real prSNPs than control prSNPs.
(EPS)

Figure S15 prSNPs distributed around the transcription start site. We assessed the location of prSNPs discovered for the real versus non-ASE predictions. We found many of the real predictions where centred around the transcription start site and downstream within the respective gene. The difference in this distribution was highly significant (*χ* ^2^ p-value<2e-16). Bins where there were significant prediction differences using a Bonferroni-corrected Fisher's exact test are highlight by stars. This result indicates that many real prSNPs are located within the gene or near promoters and suggests that the predictions are preferentially targeting regions with a higher prior of functionality.
(EPS)

Figure S16 Real prSNPs are more conserved and have lower derived allele frequency. We compared evolutionary constraint and derived allele frequency for prSNPs versus control SNPs and found that the prSNPs trended towards being slightly more conserved (PhyloP primate base conservation p = 0.12 MW; PhyloP mammalian base conservation p = 0.09; PhyloP vertebrate base conservation p = 0.07) and were more likely to have lower derived allele frequency (p = 3.437e-12 MW). This suggests that the prSNPs are more likely targeting functional sequence and are more likely to be slightly deleterious.
(EPS)

Figure S17 log2 difference from mean for Z>2 conserved singleton variants. The distribution of expression differences for conserved singleton Z>2 variants was plotted to observe the average effect size of these variants. Approximately 4/5^th^ of these variants are expected by chance. However, large differences can also be observed and are candidates for bonafide rare variants which have important effects. Of note, the CEU variants also appear enriched for expression differences relative to YRI.
(EPS)

Figure S18 Observed coincident array expression outliers by expected number for singletons. For singletons, we plot the number of expected coincident outliers at a particular Z-score (calculated by running 200 permutations of the expression individual labels) versus the observed number (black line). Where the difference is greater than 5% of all permuted observations, a red dot is marked and the number of effects per individual is tabulated. Here, we observe, consistent with results summarized in Figure 4, that YRI enrichment is more significant than the CEU. However, this plot indicates that this enrichment is across the Z-score spectrum. It should be noted that CEU SNPs are significant for Z> = 2 in this plot while the CEU SNPs did not exceed the 5% CI bound in Figure 4; this is because there we used a two-sided bound, whereas in this plot, we are using a one-sided estimate of significance since our expectation is that there will be an excess of effects. The significance for the excess for CEU SNPs is marginal though at disappears if we require the observed to be greater than 2.5% of all permuted observations.
(EPS)

Figure S19 Observed coincident RNA-seq expression outliers by expected number for singletons. For CEU RNA-Seq data we observe a similar pattern of enrichment for conserved singletons being outliers as we observe in the array data (Figure S18). Here we chose a smaller window size than we used for arrays to reduce the number of tests and increase signal (50 kb instead of 100 kb around the transcription start site). However, we find slightly fewer effects than the arrays. This is likely due to the fact we are still testing a factor of 2 more expression measurements and the RNA-Seq experiment may contain more stochasticity for any individual estimate of exon read count level. We expect that as we improve within individual estimates of specific transcript levels, we may find the RNA-Seq data will better uncover many more rare variant effects.
(EPS)

Table S1 Associated variant discovery from 1000 genomes (1KG) to HapMap 3 (HM3). EQTL variants discovered in the 1KG (best associated variant at 0.01 permutation threshold per gene) were compared to their equivalent discovery in HM3. Only 1/5 of these 1KG eQTLs were found in the HM3 and passed its equivalent significance threshold. Approximately 2% were in the HM3 but fell below this threshold indicating either genotype error in the HM3 or, less likely, stochastic improvement in association due to genotyping error in the 1KG. The remaining 4/5 of the association are for new markers that were not assayed in the HM3. This indicates that if these new variants are bonafide causal variants, whole genome sequencing is uncovering a large number that had previously not been identified. *Independent eQTLs defined by recombination interval and LD filtering as previously reported in [35].
(DOCX)

Table S2 Associated variant discovery from HapMap 3 (HM3) to 1000 genomes (1KG). EQTL variants discovered in HM3 (best associated variant at 0.01 permutation threshold per gene) were compared to their equivalent discovery in 1KG. Approximately 2/5 of the associations genotyped in both passed the equivalent discovery thresholds. Approximately 3/5 of the associations did not pass the discovery threshold in 1KG indicating that the extra multiple testing correction of the many extra variants gained through whole genome sequencing is masking some eQTLs. Only a marginal fraction of the associated SNPs were not found in the 1KG. *Independent eQTLs defined by recombination interval and LD filtering as previously reported in Nica et al. [Nica AC, Parts L, Glass D, Nisbet J, Barrett A, et al. (2011) The Architecture of Gene Regulatory Variation across Multiple Human Tissues: The MuTHER Study. PLoS Genet 7: e1002003. doi:10.1371/journal.pgen.1002003]
(DOCX)
