# Supplementary material for: Rare and Common Regulatory Variation in Population-Scale Sequenced Human Genomes
Source: PLoS Genet. 2011 Jul 21;7(7):e1002144. doi: 10.1371/journal.pgen.1002144 (PMC3141000; doi:10.1371/journal.pgen.1002144)
Supplement: Table S1 — Associated variant discovery from 1000 genomes (1KG) to HapMap 3 (HM3). EQTL variants discovered in the 1KG (best associated variant at 0.01 permutation threshold per gene) were compared to their equivalent discovery in HM3. Only 1/5 of these 1KG eQTLs were found in the HM3 and passed its equivalent significance threshold. Approximately 2% were in the HM3 but fell below this threshold indicating either genotype error in the HM3 or, less likely, stochastic improvement in association due to genotyping error in the 1KG. The remaining 4/5 of the association are for new markers that were not assayed in the HM3. This indicates that if these new variants are bonafide causal variants, whole genome sequencing is uncovering a large number that had previously not been identified. *Independent eQTLs defined by recombination interval and LD filtering as previously reported in [35]. (DOCX) [file pgen.1002144.s020.docx]

|  | Number of 0.01 eQTLs (Best association per gene) | SNP genotyped and passed discovery threshold in HM3 | SNP genotyped and did not pass discovery threshold in HM3 | SNP not found in HM3 |
| --- | --- | --- | --- | --- |
| CEU (Array) 1KG | 461 (479*) | 109 (23.6%) | 8 (1.7%) | 344 (74.6%) |
| YRI (Array) 1KG | 465 (473*) | 89 (19.1%) | 9 (1.9%) | 367 (78.9%) |
| CEU (RNA-Seq) 1KG | 971 (1072*) | 169 (17.4%) | 29 (3.0%) | 773 (79.6%) |
