# Supplementary material for: Rare and Common Regulatory Variation in Population-Scale Sequenced Human Genomes
Source: PLoS Genet. 2011 Jul 21;7(7):e1002144. doi: 10.1371/journal.pgen.1002144 (PMC3141000; doi:10.1371/journal.pgen.1002144)
Supplement: Table S2 — Associated variant discovery from HapMap 3 (HM3) to 1000 genomes (1KG). EQTL variants discovered in HM3 (best associated variant at 0.01 permutation threshold per gene) were compared to their equivalent discovery in 1KG. Approximately 2/5 of the associations genotyped in both passed the equivalent discovery thresholds. Approximately 3/5 of the associations did not pass the discovery threshold in 1KG indicating that the extra multiple testing correction of the many extra variants gained through whole genome sequencing is masking some eQTLs. Only a marginal fraction of the associated SNPs were not found in the 1KG. *Independent eQTLs defined by recombination interval and LD filtering as previously reported in Nica et al. [Nica AC, Parts L, Glass D, Nisbet J, Barrett A, et al. (2011) The Architecture of Gene Regulatory Variation across Multiple Human Tissues: The MuTHER Study. PLoS Genet 7: e1002003. doi:10.1371/journal.pgen.1002003] (DOCX) [file pgen.1002144.s021.docx]

|  | Number of 0.01 eQTLs (Best association per gene) | SNP genotyped and passed discovery threshold in 1KG | SNP genotyped and did not pass discovery threshold in 1KG | SNP not found in 1KG |
| --- | --- | --- | --- | --- |
| CEU (Array) HM3 | 473 (498*) | 241 (51.0%) | 221 (46.7%) | 11 (2.3%) |
| YRI (Array) HM3 | 489 (499*) | 219 (44.8%) | 264 (54.0%) | 6 (1.2%) |
| CEU (RNA-Seq) HM3 | 965 (1071*) | 327 (33.9%) | 626 (64.9%) | 12 (1.2%) |
